# A human epithelial cell cycle dataset

**DOI:** 10.1038/s41597-026-08216-w

**Published:** 2026-09-01

**Authors:** Rico Hiemann, Dirk Reinhold, Karsten Conrad, Stefan Rödiger, Peter Schierack, Dirk Roggenbuck

**Affiliations:** 1 Faculty Environment and Natural Sciences, Brandenburg University of Technology Cottbus-Senftenberg, Senftenberg, Germany; 2 Institute of Clinical Immunology and Cell Therapeutics, Medical Faculty, Otto-von-Guericke-University Magdeburg, Magdeburg, Germany; 3 Association for the Advancement of Immune Diagnostics, Dresden, Germany; 4 Faculty of Health Sciences Brandenburg, Brandenburg University of Technology Cottbus – Senftenberg, Senftenberg, Germany

## Abstract

We introduce a large-scale single cell dataset for cell cycle phases. The dataset includes single cell images of 4′,6-diamidino-2-phenylindole-stained human epithelioma-2 (HEp-2) cells grown on microscopic glass slides. Greyscale images of adherent HEp-2 cells were acquired at 20x magnification by an automated, inverse microscope system. Cell images were automatically segmented with 256 × 256 pixel patch size including surrounding area. Initially, 10,000 images were manually classified by experts into 5 cell-cycle groups (interphase, prophase, metaphase, anaphase, telophase) and two additional groups (artefact, triple). Based on these initial single cell-image classifications, a convolutional neural network (CNN) model (VGG16) was trained. Subsequently, additional segmented single cell images were pre-classified by the CNN and double-blinded reviewed as well as corrected by two experts to build an algorithm-aided dataset with 100,000 images. The dataset provides a resource for development of biomedical and deep learning applications and, thus, enables training and assessment of cell-cycle detection algorithms.

## Background & Summary

The detection of cell cycle-specific patterns obtained by various microscopy and imaging technologies has become central to many diagnostic applications^1–3^. The recognition and classification of mitotic figures is one of the most frequent tasks in this context in various fields such as tumor or autoimmune diagnostics. Thus, the number of mitotic figures, stained generally by hematoxylin and eosin, was reported as one of the strongest prognostic factors in tumor growth assessment^4,5^. Likewise, mitotic figures and their fluorescent staining by immune markers such as antinuclear antibodies (ANA) play a central role in the serological diagnosis of autoimmune diseases^6,7^. This applies in particular to the use of automated computer-aided diagnosis (CAD) systems^2,8,9^.

In general, the identification of mitotic cells and their classification into different mitotic phases is prone to high variability as a result of human bias, morphological variability, and different distribution of mitotic cells in tumour tissues or cell lines^10^. Thus, inter-rater agreement statistics revealed poor agreement between pathologists with Cohen’s kappa values ranging from 0.13 and 0.39 with respect to the identification of single mitotic cells in tumour tissue sections^11^. Inter-rater agreement quantifies kappa statistic in accordance with Cohen to evaluate the agreement between two classifications^12^.

Specifying mitotic patterns for ANA testing also remains a challenge, although the introduction of the first international consensus on ANA patterns (ICAP) (www.anapattern.org) in 2015 sought to improve harmonization of ANA testing by indirect immunofluorescence assay (IFA) using HEp-2 cells as substrate^13^. The mitotic patterns classified as anti-cell (AC)-24 to AC-28 require the identification of mitotic phases while the staining of the metaphase plate is essential for reporting patterns of the competent and expert levels such as AC-1 (homogenous), AC-2/30 (speckled with mitotic plate), AC-3 (centromere) or AC-29 (DNA topoisomerase I) (www.anapattern.org). Since the first attempts to use CAD-interpretation systems for ANA reporting, the recognition of mitotic figures and definition of mitotic phases either by mathematical pattern-recognition algorithms and recently machine learning have been in the limelight^14–17^.

Thus, we created an annotated scale and intensity-independent HeLa/HEp-2 cell cycle-image dataset (https://doi.org/10.5281/zenodo.18337703) for deep learning that is based on initial CNN models for biomedical research applications, which could support ANA pattern classification and tumor-associated research^18^. The dataset contains images of variable exposure times showing cells with lower and higher brightness. This allows the development of intensity-independent robust models. Each cell contains surrounding objects, which makes the set scale independent. The use of a combination of HeLa and HEp-2 images is aimed to reflect that HEp-2 cells originally thought to be derived from an epidermoid carcinoma of the larynx could be contaminated with HeLa cells based on isoenzyme analysis, HeLa marker chromosome occurrence, and DNA fingerprinting as shown for the American type culture collection (ATCC®) HEp-2 cell line CCL-23™ (www.atcc.org).

Greyscale images of 4′,6-diamidino-2-phenylindole (DAPI)-stained HEp-2 cells were acquired by an automated, inverse microscope system. Single cell images were automatically segmented with a resolution of 256 × 256 pixel. Several VGG-Net architecture CNNs were employed to learn important features for cell cycle-classification. Potential mitotic figures were identified by two immunologists [D.Ro., R.Hi.] and manually classified into 5 cell-cycle groups (interphase, prophase, metaphase, anaphase, telophase) and two additional groups (artefact, triple). Subsequently, additional images were classified by a deep learning-based pipeline (VGG16). VGG16 is a foundational CNN architecture designed for image classification tasks. It is renowned for its uniform, layer-by-layer structure, relying heavily on consecutive 3 × 3 convolution filters and max-pooling^19^. Two experts [D.Ro., D.Re.] blindly classified the annotations and reviewed the mismatched results to reach a consensus on the group assignment. This collection currently represents to the best of our knowledge the largest dataset in terms of the number of annotated mitotic figures (https://doi.org/10.5281/zenodo.18337703). Therefore, it provides researchers with new opportunities for the development and refinement of data-driven algorithms for identifying mitotic figures on slides and microtiter plates as shown recently for AI-based pattern interpretation of ANA image analysis^20^. This dataset could also be applied to the segmentation of cells in fluorescence tissue staining when the detection of cell nuclei is of interest.

## Methods

### Selection and preparation of samples

Immunofluorescence images of HEp-2 cell preparations on glass slides stained with DAPI were obtained from the diagnostic archive of the Association for the Advancement of Immune Diagnostics (Dresden, Germany). The samples originated from 10 years of measurement of consecutive HEp-2 preparations as part of routine diagnostics for the detection of ANA.

Images of wells on the slide were taken with an inverted fluorescence microscope (akironNEO Medipan, Dahlewitz, Germany) in one focal plane per image at a magnification of 200 (Data acquisition parameters: camera model: Basler AC1920-40; sensor: Sony IMX249 CMOS, sensor size: 1/1,2”; image format: uint8, 1936 × 1216, no binning, saved as single channel webp; pixel size: 0.46510 µm/pixel; objective: Plan N 20x/0.4; exposure time: Basler, autoadjusted to staining intensity, range of 30–100 ms; light source: LED 400 nm; filter: triple-band, excitation: 382/473/630, dichroic mirror: 430/529, emission: 405/495/655)^8,21^. Cell segmentation was performed using Otsu’s thresholding as implemented in the akironNEO® software, a widely used and well-established method for separating foreground objects from background in bioimage informatics workflows^22^.

### Initial manually annotated dataset by experts - expert dataset (ExpD)

**Fig. 1:**
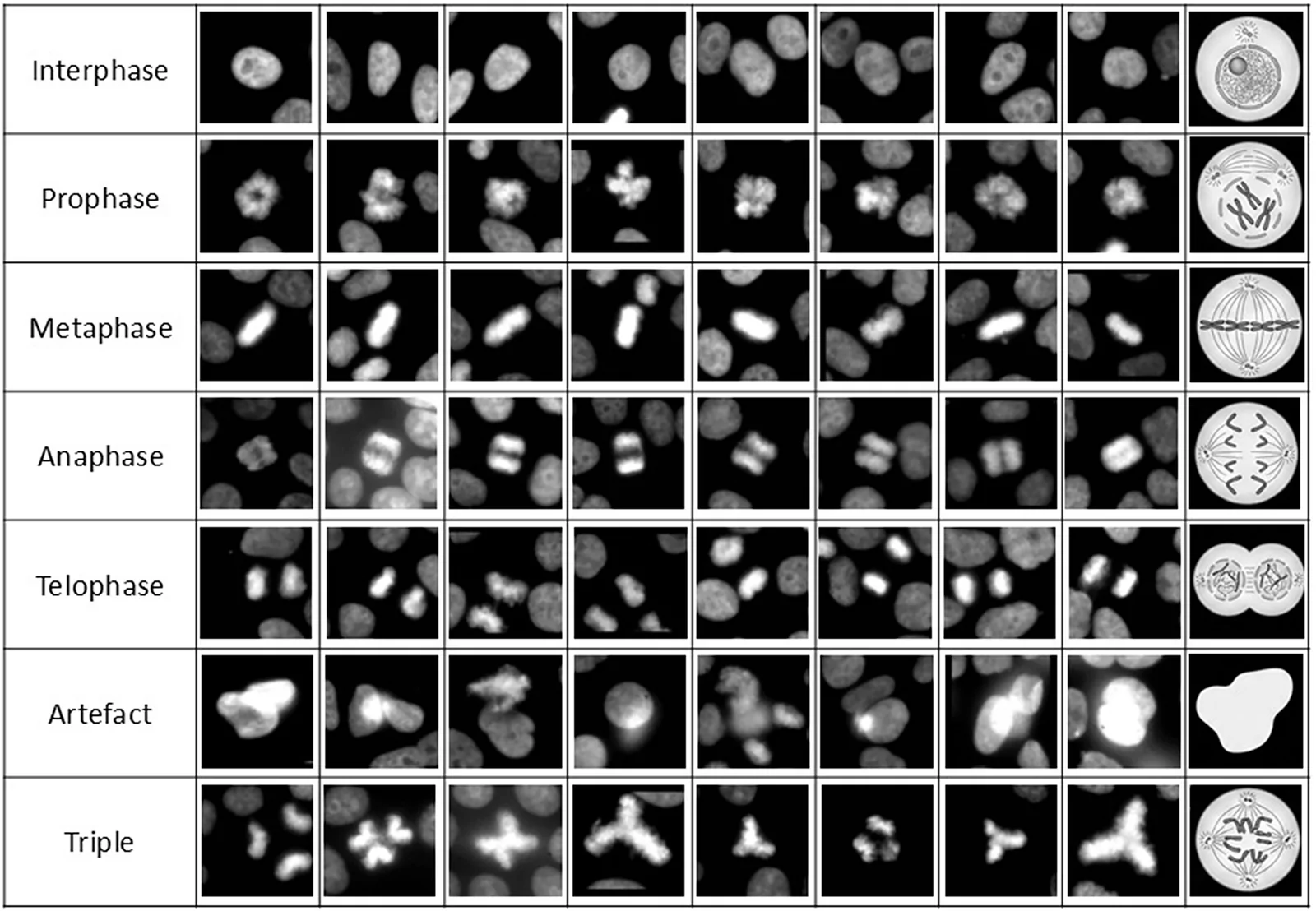
Image examples for the 5 cell cycle phases annotated in the data set (https://doi.org/10.5281/zenodo.18337703) and grouped accordingly^18^. Additionally, ambiguously centered cells and overlapping or occluded structures are annotated as artefacts and assigned to one group. Furthermore, triple structures, which occur as a result of cell division errors and are a common feature of cancer cells, were assigned to the Triple group. The last column shows schematic images of the 5 cell cycle phases.

Primary annotations were performed by two experts trained in the field of immunology [R.Hi., D.Ro.]. The experts classified the stained images based on previously reported DAPI image data of mitotic figures into the following groups (Fig. 1)^23^:

1. **Interphase:** Rounded nucleus of mostly homogeneous texture. In individual cases, nucleoli can be seen in the nucleus. Interphase nuclei are characterized by less condensed chromatin, resulting in low intensity staining and larger nuclei compared to the cell division phases.
2. **Prophase:** Clearly condensed chromatin with an irregular texture, which leads to an increased intensity of the nucleus with smaller nucleus size compared to interphase cells. Prophase nuclei have a rounded shape with distinct corners and edges around the rim.
3. **Metaphase:** Narrow, linear shape with very intense staining due to highly condensed chromatin in the metaphase plate. Homogeneous to slightly granular texture with some slight corners and edges in the rim.
4. **Anaphase:** Rounded to rectangular, mostly symmetrical shape with high intensity and recognizable separation zone in the middle area. Protein filaments are partially visible, which pull the chromatin towards the poles.
5. **Telophase:** Mostly symmetrical, parallel, already separated daughter cells with similar shapes and highly condensed chromatin with intense staining. The stained chromatin is usually smaller than in metaphase.
6. **Artefact:** Unassignable objects due to detached cells, strong overlap or occlusion of cells during cell culture. Other fluorescent objects that cannot be assigned to any other group.
7. **Triple:** Cells with discernible division defects, especially in metaphase and telophase. In metaphase, more star-shaped with 3 arms than linearly elongated. In telophases, three instead of two identical daughter cells of similar size. Objects caused by division errors and polyploidy of the tumor cell line.

The group of artefacts plays a special role in this context. This group was originally used for images that do not exhibit mitotic figures but cannot be clearly assigned to interphase cells either.

**Fig. 2:**
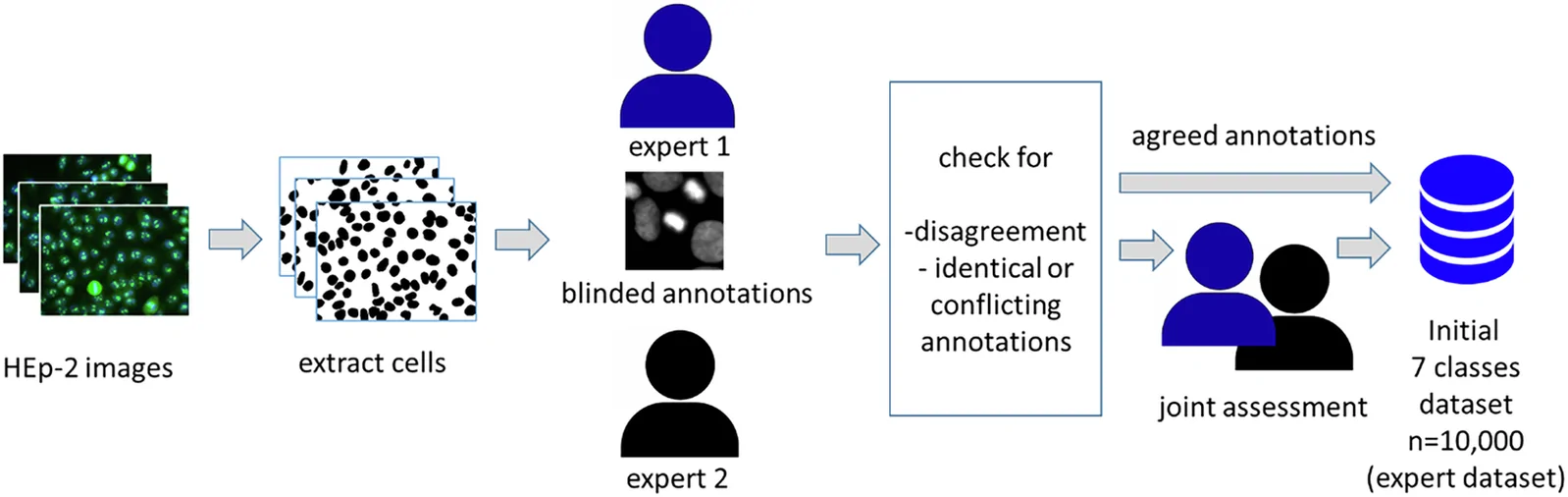
Creation of the initial manually labelled expert-dataset variant, which is the base for the final CNN-expert data set. Every 20x microscope image was screened automatically for brightest objects to enrich cells of interest. The first and second experts were able to view segmented cell images showing the segmented object in the center and the surrounding objects.

Cells with disagreement were re-assigned to both experts for a common consensus. Both experts performed blinded annotations not knowing cell-class decisions of the other expert (Fig. 2). The annotation algorithm provides a mode for annotating cells that allows the expert to evaluate the annotation of cells with unknown class. After selecting the respective classes, the next random annotation(s) was presented. The resulting confusion matrix, along with the corresponding global metrics, is given in Suppl. Table 1.

It is well known that the concordance of different experts in assessing mitotic figures is not perfect. In all cases where both experts could not agree on the same classification encompassing several doubtful candidates, both experts re-evaluated them to find agreement on the group. The re-evaluation resulted in the manually labeled dataset (ExpD) variant encompassing 2,500 interphase cell images and 1,250 images each for the remaining groups (Table 1). To lower the risk of missing labels, which tend to happen with manual annotations of large datasets, we used an algorithmic-assisted pipeline to grow our annotated dataset by a factor of ten.

**Table 1:** Number of images assigned to the 7 classification groups and corresponding file numbers and data sizes in the expert (ExpD) and enlarged datasets (CNN-D).

Classification groups	ExpD	CNN-D
Interphase	2,500	28,639
Prophase	1,250	6,604
Metaphase	1,250	31,582
Anaphase	1,250	3,245
Telophase	1,250	20,941
Artefact	1,250	6,468
Triple	1,250	2,521
**Total file number**	10,000	100,000
**Size**	135 MB	2,960 MB

### Enlarged dataset for mitotic figures (CNN-D)

To improve the quality of our dataset, we used a deep learning image classifier based on CNNs trained on the manually labeled ExpD dataset. Based on these data, our primary goal was to improve the separation of similar mitotic figures belonging to sequential mitotic phases, e.g., the transition from prophase to metaphase and from anaphase to telophase.

For cell classification, we used a standard CNN architecture based on VGG16 as a backbone. We trained this network for 25 cycles using the ExpD dataset, utilizing 256 × 256-pixel image slices around the annotated cells in the dataset for each cycle. Predicted cell classifications by the network were reviewed again by both experts to account for potentially misclassified images. Afterwards, the trained VGG16 network was used to pre-classify new images of the same size (n = 100,000).

**Fig. 3:**
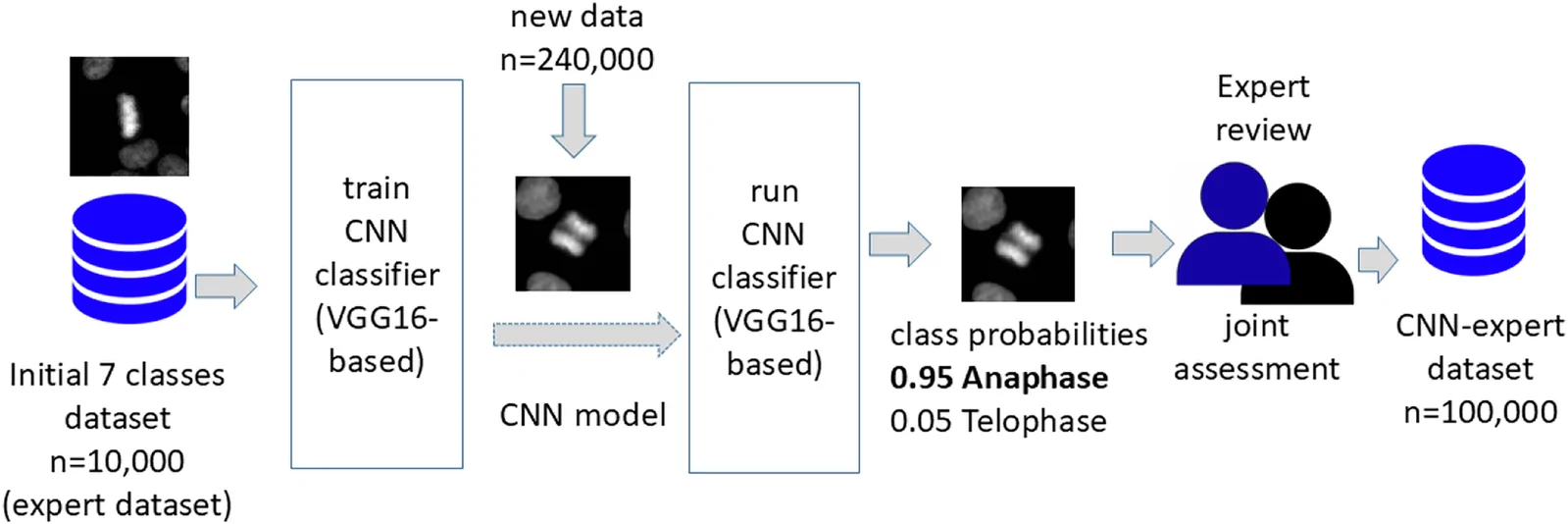
Neural network-aided classification of additional images for the enlarged dataset CNN-D (CNN + expert labelled sets).

The goal of this approach was to eliminate the bias and improve the entire dataset using a deep learning image classifier based on CNNs. In this context, a deep network suggests additional potential candidates for mitotic figures, which would need to be evaluated by human experts and assigned to the various classification groups in our dataset (Fig. 3). Eventually, the human experts reviewed the pre-classified images and jointly assigned them to the appropriate groups (Table 1). The resulting confusion matrix, along with the corresponding global metrics, is presented in Suppl. Table 2.

## Data Records

We provide the original images in WebP format on Zenodo (https://doi.org/10.5281/zenodo.18337703)^18,24^. All images have been fully anonymized. WebP was used because it maintains a good human-perceivable visual quality and superiority over PNG in lossless image compression^25^. We use lossless WebP to preserve exact pixel values while benefiting from compact file sizes, which reduces disk usage and accelerates data loading for machine learning training and evaluation^26,27^. Each filename contains the group name assigned by experts followed by a unique image number per group (e.g., Interphase_00010) or “DAPI” followed by a unique image number for overall microscopic images.

## Technical Validation

Our technical validation of the dataset was performed at two levels: First, we evaluated the quality of the assigned labels by performing a classification experiment of mitotic figures versus other cells. Second, we performed a class recognition task on the complete test set. Both are informative and address different questions: While the first test provides information on how well class separation is possible and, thus, indirectly assesses the quality of label classes, the second test assesses the coverage of mitotic figures in the CNN-D. Accuracy was used as measuring criterion, defined as ratio of the number of correctly predicted images to the total number of evaluated images (Suppl. Tables 1-2).

### Classification of preselected cells

For this validation task, 256 × 256 pixel patches containing individual cells of all classes in the center of the image patch except ambiguous cells (mitotic figure) were segmented from the CNN-initial variant of the dataset. We used a state-of-the-art CNN classification network based on a non-pretrained VGG16. The network was trained for a cycle of 25 epochs with a maximum learning rate of 10^−6^ and the Lion optimizer. Using this approach, we achieved an accuracy (proportion of correctly predicted images to the total number of images evaluated) of 92.0% with the test set. The main confusion relates to adjacent mitotic figures, while all other cell types were well separated by the classifier. This result is also consistent with the high intra- and inter-rater variance on this task by human experts.

## Supplementary information


Supplemental Material


## Data Availability

The dataset is available at https://zenodo.org/records/18337703^18^.

## References

[1] Bertram, C. A., Aubreville, M., Marzahl, C., Maier, A. & Klopfleisch, R. A large-scale dataset for mitotic figure assessment on whole slide images of canine cutaneous mast cell tumor. *Sci Data* **6**, 274 10.1038/s41597-019-0290-4 (2019). 31754105 PMC6872565

[2] Cinquanta, L., Bizzaro, N. & Pesce, G. Standardization and Quality Assessment Under the Perspective of Automated Computer-Assisted HEp-2 Immunofluorescence Assay Systems. *Frontiers in Immunology* **12**, 1–8 (2021). 10.3389/fimmu.2021.638863 PMC7947926 33717188

[3] Aubreville, M. *et al*. A comprehensive multi-domain dataset for mitotic figure detection. *Sci Data* **10**, 484 10.1038/s41597-023-02327-4 (2023). 37491536 PMC10368709

[4] Elston, C. W. & Ellis, I. O. Pathological prognostic factors in breast cancer. I. The value of histological grade in breast cancer: experience from a large study with long-term follow-up. *Histopathology* **19**, 403–410 10.1111/j.1365-2559.1991.tb00229.x (1991). 1757079

[5] Romansik, E. M., Reilly, C. M., Kass, P. H., Moore, P. F. & London, C. A. Mitotic index is predictive for survival for canine cutaneous mast cell tumors. *Vet Pathol* **44**, 335–341 10.1354/vp.44-3-335 (2007). 17491075

[6] Agmon-Levin, N. *et al*. International recommendations for the assessment of autoantibodies to cellular antigens referred to as anti-nuclear antibodies. *Ann Rheum Dis* **73**, 17–23 10.1136/annrheumdis-2013-203863 (2014). 24126457

[7] Mahler, M., Meroni, P. L., Bossuyt, X. & Fritzler, M. J. Current concepts and future directions for the assessment of autoantibodies to cellular antigens referred to as anti-nuclear antibodies. *J Immunol Res* **2014**, 315179 10.1155/2014/315179 (2014). 24868563 PMC4020446

[8] Willitzki, A. *et al*. New platform technology for comprehensive serological diagnostics of autoimmune diseases. *Clinical and Developmental Immunology* **2012**, 284740 (2012). 23316252 10.1155/2012/284740 PMC3536031

[9] Mayr, S. *et al*. Pilot Study of AI-Assisted ANA Immunofluorescence Reading-Comparison with Classical Visual Interpretation. *J Clin Med* **14** 10.3390/jcm14196924 (2025). PMC12525240 41096004

[10] Meyer, J. S. *et al*. Breast carcinoma malignancy grading by Bloom-Richardson system vs proliferation index: reproducibility of grade and advantages of proliferation index. *Mod Pathol* **18**, 1067–1078 10.1038/modpathol.3800388 (2005). 15920556

[11] Malon, C. *et al*. Mitotic figure recognition: agreement among pathologists and computerized detector. *Anal Cell Pathol (Amst)* **35**, 97–100 10.3233/ACP-2011-0029 (2012). 21965283 PMC4605539

[12] Cohen, J. A coefficient of agreement for nominal scales. *Educational and Psychological Measurement* **20**, 37–46 (1960).

[13] Tebo, A. E. *et al*. The antinuclear antibody HEp-2 indirect immunofluorescence assay: a survey of laboratory performance, pattern recognition and interpretation. *Auto Immun Highlights* **12**, 4 10.1186/s13317-020-00146-w (2021). 33640027 PMC7916270

[14] Hiemann, R. *et al*. Challenges of automated screening and differentiation of non-organ specific autoantibodies on HEp-2 cells. *Autoimmun Rev* **9**, 17–22 10.1016/j.autrev.2009.02.033 (2009). 19245860

[15] Cascio, D., Taormina, V. & Raso, G. Deep Convolutional Neural Network for HEp-2 Fluorescence Intensity Classification. *Appl Sci* **9**, 408 10.3390/app9030408 (2019).

[16] Wu, Y. D. *et al*. Application of Supervised Machine Learning to Recognize Competent Level and Mixed Antinuclear Antibody Patterns Based on ICAP International Consensus. *Diagnostics (Basel)* **11**, 642 10.3390/diagnostics11040642 (2021). 33916234 PMC8066559

[17] Weiss, R. *et al*. Applications of Neural Networks in Biomedical Data Analysis. *Biomedicines* **10**, 1–32 10.3390/biomedicines10071469 (2022). PMC9313085 35884772

[18] Hiemann, R. *et al*. The human epithelial cell cycle dataset for deep learning and biomedical research (HEp-2 dataset) [Data set]. *Zenodo*. 10.5281/zenodo.18337703 (2026).

[19] Simonyan, K. & Zisserman, A. Very Deep Convolutional Networks for Large-Scale Image Recognition, arXiv:1409.1556 (2015).

[20] Schmidt, J. *et al*. Comparison of manual with artificial intelligence-aided interpretation of ANA HEp-2 IIF assay patterns in a clinical diagnostics lab. *Clin Chim Acta* **31**(584), 120881 10.1016/j.cca.2026.120881 (2026). 41628845

[21] Egerer, K. *et al*. Automated evaluation of autoantibodies on human epithelial-2 cells as an approach to standardize cell-based immunofluorescence tests. *Arthritis Res Ther* **12**, R40 10.1186/ar2949 (2010). PMC2888187 20214808

[22] Schneider, J. *et al*. Open source bioimiage informatics tools for the analysis of DNA damage and associated biomarkers. *J Lab Precis Med* **4**, 21 10.21037/jlpm.2019.04.05 (2019).

[23] Van Hooser, A. A., Yuh, P. & Heald, R. The perichromosomal layer. *Chromosoma* **114**, 377–388 10.1007/s00412-005-0021-9 (2005). 16136320

[24] Ginesu, G., Pintus, M. & Giusto, D. D. Objective assessment of the WebP image coding algorithm. *Signal Processing: Image Communication* **27**, 867–874 10.1016/j.image.2012.01.011 (2012).

[25] Hu, J., Song, S. & Gong, Y. Comparative performance analysis of web image compression. *10th International Congress on Image and Signal Processing, BioMedical Engineering and Informatics (CISP-BMEI)*. 1–5 10.1109/CISP-BMEI.2017.8301939 (2017).

[26] Huszar, F., Theis, L., Shi, W. & Cunningham, A. Lossy Image Compression with Compressive Autoencoders. *Apollo - University of Cambridge Repository.* 10.17863/CAM.51995 (2020).

[27] Johnston, N. *et al*. Improved lossy image compression with priming and spatially adaptive bit rates for recurrent networks. *IEEE/CVF Conference on Computer Vision and Pattern Recognition*. 4385–4393 (2018).

