# Supplementary material for: A human epithelial cell cycle dataset
Source: Sci Data. 2026 Sep 1;13:1261. doi: 10.1038/s41597-026-08216-w (PMC13534591; doi:10.1038/s41597-026-08216-w)

Suppl. Tab. 1: Global metrics and confusion matrix VGG16 model of the expert dataset (ExpD).

accuracy: 92.00%; validation loss: 0.2827; macro precision: 0.9606; macro recall: 0.9578; macro F1-Score: 0.9580

weighted precision: 0.9635; weighted recall: 0.9623; weighted F1-Score: 0.9619

precision, recall, f1-score, and support of anaphase figures (0.99, 0.88, 0.93, 1250, respectively), artefact figures (0.96, 0.89, 0.92, 1250, respectively), interphase figures (0.98, 0.99, 0.99, 2500, respectively), metaphase figures (0.99, 0.98, 0.98, 1250, respectively), prophase figures (0.96, 0.99, 0.97, 1250, respectively), telophase figures (0.90, 0.99, 0.94, 1250, respectively), and triple figures (0.94, 0.99, 0.96, 1250, respectively)

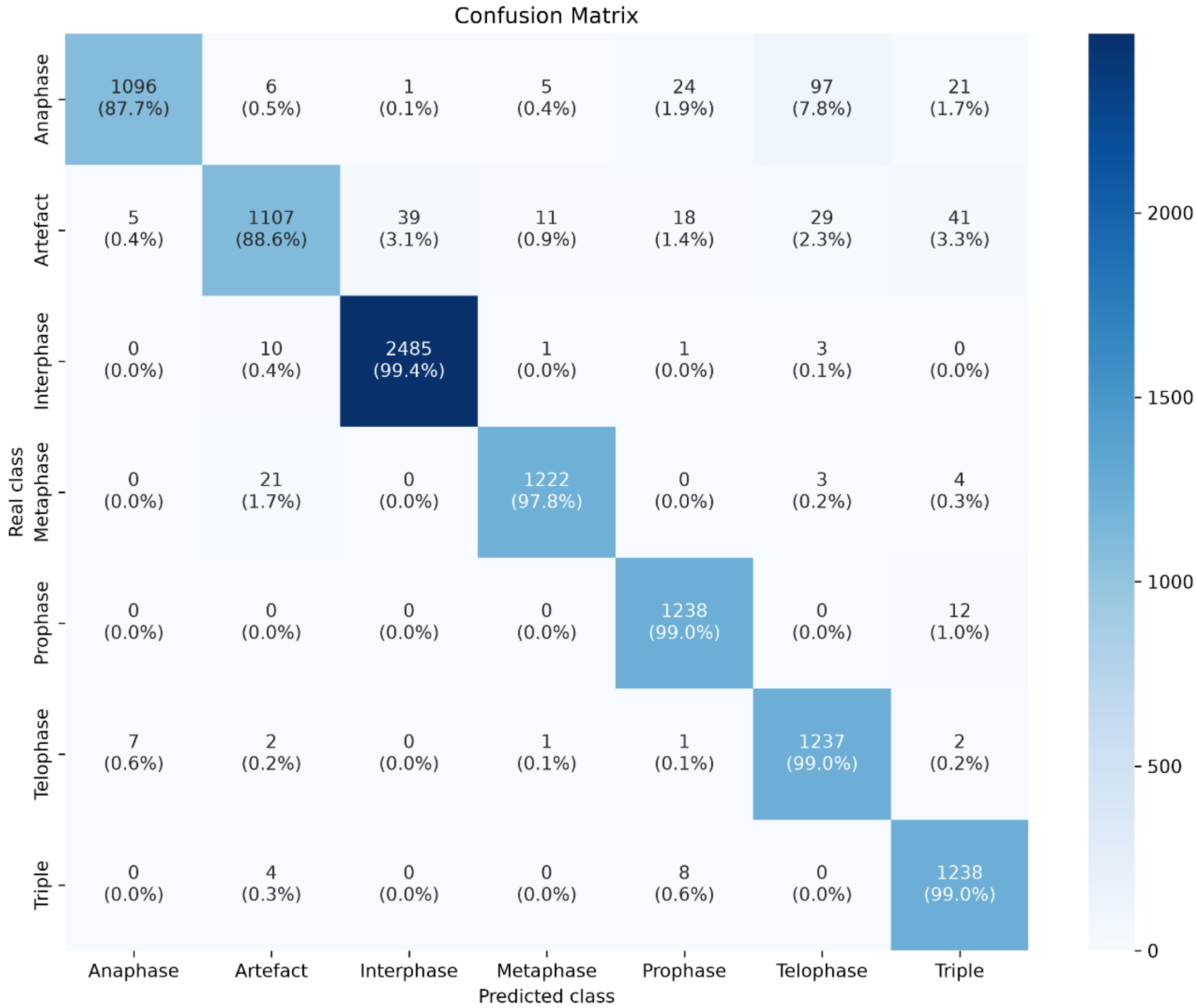

Suppl. Tab. 2: Global metrics and confusion matrix VGG16 model of the enlarged dataset (CNN-D).

accuracy: 81.82%; validation loss: 0.8163; macro precision: 0.7192; macro recall: 0.8098; macro F1-Score: 0.7376

weighted precision: 0.8651; weighted recall: 0.8182; weighted F1-Score: 0.8315

precision, recall, f1-score, and support of anaphase figures (0.67, 0.81, 0.73, 3245, respectively), artefact figures (0.40, 0.69, 0.51, 6468, respectively), interphase figures (0.95, 0.97, 0.96, 28639, respectively), metaphase figures (0.94, 0.78, 0.85, 31582, respectively), prophase figures (0.83, 0.78, 0.81, 6604, respectively), telophase figures (0.88, 0.71, 0.78, 20941, respectively), and triple figures (0.36, 0.93, 0.52, 2521, respectively)

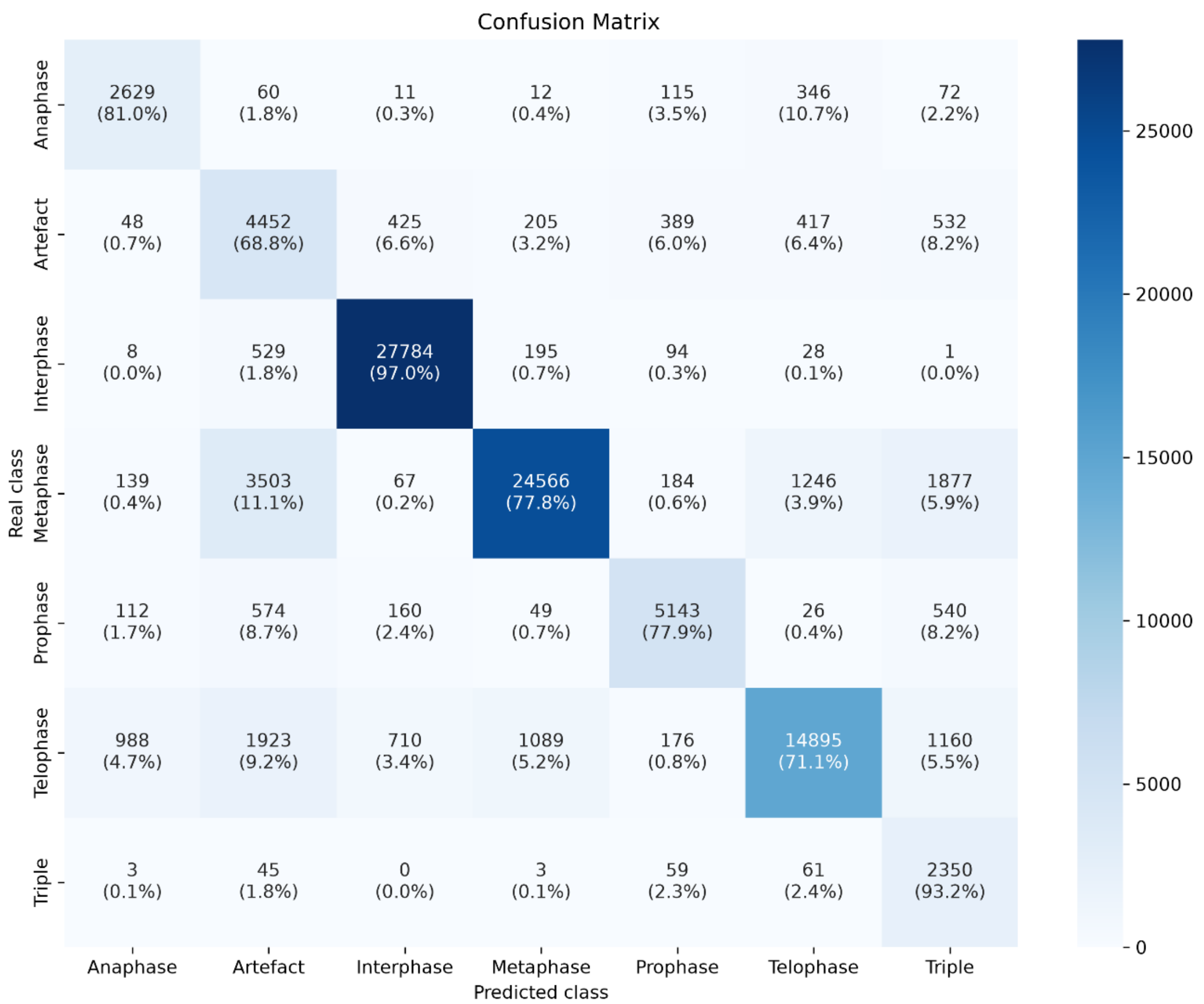

Supplement: Supplementary file 1 [file 41597_2026_8216_MOESM1_ESM.pdf]
